# Application of Multi-State Model in Analyzing of Breast Cancer Data

**Published:** 2020-01-05

**Authors:** Mahtab Vasheghani Farahani, Parisa Ataee Dizaji, Hamid Rashidi, Fariborz Mokarian, Akbar Biglarian

**Affiliations:** ^1^Department of Biostatistics, University of Social Welfare and Rehabilitation Sciences, Tehran, Iran; ^2^Faculty of Medicine, Cancer Prevention Research Center, Isfahan University of Medical Sciences, Iran; ^3^Department of Biostatistics, Social Determinants of Health Research Center, University of Social Welfare and Rehabilitation Sciences, Tehran, Iran

**Keywords:** Survival analysis, Multistate model, Breast cancer

## Abstract

**Background:** The multistate model is used generally to fit the longitudinal data. This model can determine the natural trend of disease progress in different states of treatment, recuperate, metastasis and finally death. We aimed to use multistate models in order to analyzing breast cancer (BC) data.

**Study design:** A historical cohort study.

**Methods:** In this historical cohort study, 573 women with BC were studied. These patients were referred to Isfahan Sayed-o-Shohada Hospital during 1999-2006 and followed up to Apr 2017. The corresponding provided data were gathered by Isfahan Cancer Prevention Center. Then data analyzed by multistate models in R 3.4.1 software.

**Results:** The mean and standard deviation of women age were 47.19±10.77 years. The transition probability from state of first treatment to recuperate state was 71%, to metastasis state 2% and to death was 16%. The sojourn time in different states of disease was 2.39 yr for first treatment, 6.93 yr for recuperate and 0.16 yr for death.

**Conclusion:** This model is able to predict the transition probabilities in different state of disease, so its results are useful for clinical researches. In addition, with transition probabilities and also survival mean in each state in hand, the physicians will be able to suggest suitable treatment plans for patients.

## Introduction

Cancer is a set of diseases which has uncontrolled increasing in physical size and also extension of abnormal cells ^1^. Cancers are not limited to any specific time or place and are the second leading cause of death in the world ^2^. Moreover, as non-communicable disease, after cardiovascular diseases, cancer is the most critical health problem around the world. There are multiple various complications around it, it is notably a costly burden and still the response to treatment is unsatisfactory and incomplete in many cases ^3^, so that, the increase of global burden of cancer in less developed country is more than other country^4^. Based on WHO reports, cancer is the first or second leading cause of death in many countries ^5^. In 2018, 18.1 million cancer cases and 9.6 million cases of death due to cancer were estimated from around the world ^6^. In 2015, breast, lung and colorectal cancers were the most common cancers ^5^ and in 2018, breast, colorectal and lung cancers were the most commonly diagnosed cancers ^6^. Breast cancer (BC) is the most common type of cancer diagnosed in women and is the main cause of cancer-related death in females in 2015 ^5^ and also in 2018 ^6^. This cancer is a serious health problem for women in the world and about 1.67 million women diagnosed with BC every year globally ^7^.

BC is the most common type of cancer after skin cancer in Iran. It was reported 8090 new patients are recognized with this disease yearly and more than 1300 death occurs due to BC ^8^. In addition, about 21% of all reported cases of cancer in women are related to malignant BC ^9^. Unfortunately, about 70% of Iranian women seek medical treatment in advanced stages of the disease in which the treatment options are very limited and mostly ill-fated ^10^. In Iran, the age-standardized rates for BC and for mortality were reported 33.21 and 14.2 years per 100,000, respectively ^11^. In recent years the prevalence of the disease shows a growing trend and the 5 and 10 yr survival rate of patients was reported 88% and 80%, respectively ^12^.

Relapse and death of BC are usually among noted events for researchers in survival analysis of long-term disease, in which the recurrence of the disease consider as a recurring event and death consider as a final event ^13^. In addition, due to the transition from the early stages, which usually occurs after surgery or beginning of treatment, to terminal state or endpoint, transition probabilities can be estimated through statistical analysis ^14^. In some clinical studies, more than one endpoint can be defined for a specified event. For example, in BC survival without disease, recovery, metastasis or death can be considered as the endpoints ^13, 14^. In such cases, a model of competitive risk or a multi-state model is used. In a multi-state model the main focus is on moving from one state to another ^14^. Indeed, multi-state models are used to analyze complex time to event problems with multiple endpoints ^15^. These models permit for qualification of risk factors and description of intermediate events and all pathways in the analysis of multi-state data ^16^.

These models actually are generalization of generalized linear models and they work very efficiently when it comes to modeling the longitudinal data with dependency between observations ^17, 18^. We aimed to use the multi-state model to analyze the BC data.

## Methods

This study was a historical cohort study and enrolled 633 participants. Women with clinically approved BC referred to Seyed-o-Shohada Hospital in Isfahan, Iran from 1999 to 2006 and followed up to Apr 2017.

All of subjects received at least one treatment including surgery, chemotherapy, hormone therapy and radiotherapy at the time of study. Independent variables used in this study were age, tumor size, the number of lymph nodes, and the number of involved lymph nodes on the number of removed lymph nodes, estrogen receptor status, progesterone receptor status, human epidermal growth hormone receptor status and hormone P53 receptor status.

The research data were collected from subjects’ medical records at the mentioned hospital and also with assistance of the Isfahan Cancer Prevention Research Centre. Patients, who experienced only one condition and were censored for some reason, were excluded from this study.

**Figure 1:**
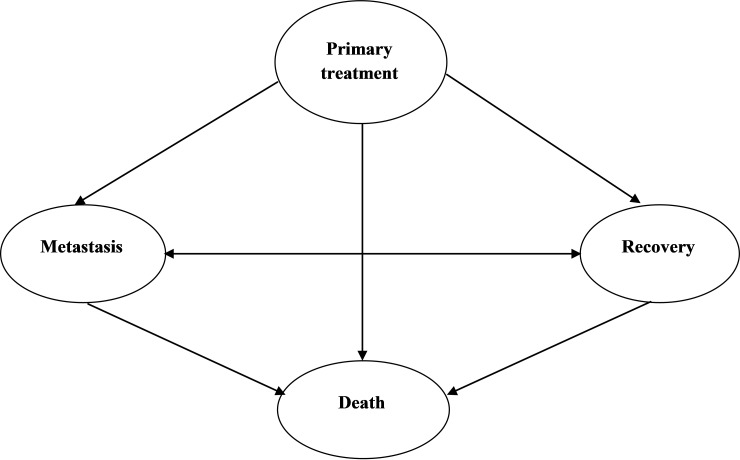
Allowed transition paths for multi-state model in Breast cancer

Overall, 573 patients were studied as a sample. These patients were followed from the beginning of initial treatment to Apr 2017 in terms of subject’s state transition between available states in the study (Figure 1). These states were initial treatment as the first state, recovery as the second state, metastasis as the third state and death as the absorbent or terminal state. Therefore each patient will experience at least one of the metastasis, recovery or death state with a transitional probability after receiving initial treatment. As shown in Figure 1 for all of subjects moving from first state to any other state is possible, but metastasis and recovery states are accessible by each other and both can end to terminal state (death).

Markov's continuous-time model fitted to data and then maximum likelihood method was used to estimate the parameters of the model. In this scene, tdc.msm library was used to assess the Markov property. Afterward, hazard ratio was calculated by exponentiation the estimated effects of the independent variable on the logarithm of the transition rates (i.e. exp (b)). If the ratio is 1 that means that the risks are the same. If it is greater than 1, then the risk is higher, and vice versa ^19^. Moreover, the average survival time in each states estimated by reciprocal of arrays on main diagonal of transition intensity matrix ^20^. The analysis of data was performed by MSM package in R software version 3.4.1.

## Results

Overall, 573 females with BC were studied with an average age 47.19±10.77 yr and the median age 46 years. The youngest and oldest subjects included in the study were respectively 23 and 75 yr old.

Thirteen deaths occurred in the initial treatment, 31 deaths occurred in recovery status and 146 deaths occurred in metastasis status (Table 1).

**Table 1:** Distribution of patients with BC in various states of disease in transition matrix

**Patient condition**	**Initial treatment**	**Recovery**	**Metastasis**	**Death**
Initial treatment	983	516	43	13
recovery	0	318	125	31
metastasis	0	5	7	146

Initial rough values for transition intensities were calculated regarding the initial transition density and matrix. Then the multi-state model was fitted to the dataset without considering independent variables. The estimated transition probability matrix was calculated applying Markov multi-state model for a one-year interval (Table 2). This table provides important clinical insights on disease progression expectancy. A patient with BC in the initial state would recover successfully with a probability of 33%, and there was notable lower probability of 2% for developing metastasis and 3% to die over a year (Table 2).

**Table 2:** The one-year transition probability matrix confidence interval for BC data

**Variables**	**Initial treatment**	**Recovery**	**Metastasis**	**Death**
Initial treatment	0.626 (0.131, 0.646)	0.326 (0.281, 0.752)	0.018 (0.013, 0.027)	0.031 (0.035, 0.208)
Recovery	0.000 ( - )	0.935 (0.448, 0.944)	0.025 (0.011, 0.036)	0.040 (0.035, 0.542)
Metastasis	0.000 ( - )	0.875 (0.404, 0.898)	0.023 (0.011, 0.034)	0.102 (0.074, 0.589)
Death	0.000 ( - )	0.000 ( - )	0.000 ( - )	1.000 ( - )

Considering a 5 year period of time, a patient who is in the initial state of treatment would recover with a probability of 71%, would developed metastasis with a probability of 2% and would die with a probability of 17% (Table 3).

**Table 3:** The five-year transition probability matrix for BC data

**Variables**	**Initial treatment**	**Recovery**	**Metastasis**	**Death**
Initial treatment	0.096	0.715	0.020	0.169
Recovery	0.000	0.789	0.021	0.190
Metastasis	0.000	0.639	0.019	0.242
Death	0.000	0.000	0.000	1.000

Variable dependency can be effective at states transition rates, the multi-state model was fitted with independent variables this time. The multi-state model which fitted with independent variables was significantly more suitable than model without independent variables (*P*< 0.001). Therefore, the Markov multi-state model with independent variables was fitted to the data and hazard ratio were estimated (Table 4). As the age increases, the risk of death for a patient who is in the initial treatment decreases 10% after adjustment for other variables and also the risk of death for those who in metastasis state increases 12%. likewise with increasing tumor size and other variables kept fixed, the risk of moving from the first state to second state (metastasis) increases 84% and the risk of death for those in initial state treatment increases to 3.45 times and also the related risk for developing metastasis for patients in recovery state increases 12%.

**Table 4:** The estimated hazard ratios from multi-state model with independent variable

**Transition paths for independent variables**	**Hazard ratio (95% CI)**
Age of patient	
Initial treatment- recovery	1.026 (0.998, 1.056)
Initial treatment- metastasis	0.913 (0.858, 0.972)
Initial treatment- death	0.903 (0.756, 1.077)
Recovery- metastasis	0.961 (0.921, 1.002)
Recovery- death	0.762 (0.569, 1.020)
Metastasis- recovery	1.029 (0.981, 1.079)
Metastasis- death	1.121 (1.083, 1.161)
Tumor size	
Initial treatment- recovery	1.700 (1.338, 2.159)
Initial treatment- metastasis	1.835 (0.578, 5.822)
Initial treatment- death	3.453 (1.035, 11.526)
Recovery- metastasis	1.122 (0.210, 5.983)
Recovery- death	0.004 (0.003, 0.005)
Metastasis- recovery	1.007 (0.445, 2.276)
Metastasis- death	1.397 (1.110, 1.757)
Number of involved/removed lymph nodes	
Initial treatment- recovery	2.551 (1.437, 4.530)
Initial treatment- metastasis	0.693 (0.193, 2.482)
Initial treatment- death	0.021 (0.000, 1.120)
Recovery- metastasis	0.009 (0.001, 0.077)
Recovery- death	0.082 (0.014, 0.474)
Metastasis- recovery	0.000 (0.000, 0.000)
Metastasis- death	3.580 (1.174, 10.920)
Progesterone receptor status	
Initial treatment- recovery	0.867 (0.498, 1.510)
Initial treatment- metastasis	0.679 (0.243, 1.892)
Initial treatment- death	0.004 (0.003, 0.005)
Recovery- metastasis	0.678 (0.471, 0.974)
Recovery- death	0.009 (0.007, 0.011)
Metastasis- recovery	2.059 (0.384, 11.023)
Metastasis- death	1.261 (0.824, 1.929)
Estrogen receptor status	
Initial treatment- recovery	1.135 (0.888, 1.450)
Initial treatment- metastasis	0.506 (0.201, 1.276)
Initial treatment- death	0.557 (0.248, 1.249)
Recovery- metastasis	0.781 (0.532, 1.144)
Recovery- death	0.002 (0.000, 0.007)
Metastasis- recovery	0.286 (0.068, 1.208)
Metastasis- death	0.465 (0.364, 0.594)
Human epidermal growth hormone receptor status (HER2)
Initial treatment- recovery	0.862 (0.512, 1.453)
Initial treatment- metastasis	1.233 (0.260, 5.850)
Initial treatment- death	1.786 (0.018, 33.379)
Recovery- metastasis	0.414 (0.109, 1.578)
Recovery- death	0.220 (0.008, 6.113)
Metastasis- recovery	0.633 (0.169, 2.373)
Metastasis- death	2.180 (1.131, 4.204)
Hormone P53 receptor status	
Initial treatment- recovery	0.872 (0.491, 1.548)
Initial treatment- metastasis	2.474 (0.689, 8.879)
Initial treatment- death	0.069 (0.008, 0.590)
Recovery- metastasis	1.119 (0.592, 2.116)
Recovery- death	1.738 (0.676, 4.470)
Metastasis- recovery	1.043 (0.522, 2.083)
Metastasis- death	1.909 (0.841, 4.331)

The average sojourn time of patients was calculated in each state for BC patients. The maximum mean sojourn time considering the influence of independent variables related to recovery was equal to 6.93 yr (Table 5).

**Table 5:** Mean sojourn time (in years) estimation for BC data using multi-state model

**Variables**	**Without independent variable**	**With independent variable** ^a^
Initial treatment	2.135 (1.899, 2.400)	2.390
Recovery	1.546 (0.791, 3.020)	6.934
Metastasis	0.041 (0.023, 0.071)	0.157

^a^ In this case, CI is not calculable by the sojourn.msm function

## Discussion

Local recurrence is common for BC patients ^21^ and these patients have probably metastasis and consequently have worse survival ^22^. Occurrence of these relapses or death event may be described by prognostic factors such as tumor biological sizes or other related bio-properties. Therefore, a model should be used to consider the heterogeneity in these kinds of data ^12^. The multi-state analysis is a suitable method to analyze the data with complex patterns of variability maintaining focus on hierarchical sources ^23^. Estimation of transition rate between disease states, assessing the effect of risk factors on possible transitions and investigating the effect of medical interventions are the encouraging advantages provided by these models ^24^. Constructing multistate models provide an extensive view of the disease progression and enable us to estimate the number of individuals who will be in the various states at times in the future ^25^. This form of modeling can also be used for health care evaluation too. These include evaluating the costs and clinical implications in chronic disease ^26^. The number of involved lymph nodes and tumor size were the important clinical factors of patient status ^8^. Tumor size, lymph node status, estrogen receptor status (ER) and human epidermal growth hormone receptor status (HER2) are the contributing factors in improving the prognosis of BC ^7^. Tumor characteristics and number of involved lymph nodes increase the death rates in BC patients using Cox and Frailty models ^27^. In another study, disability model for BC showed that age and number of involved lymph nodes had significant effect on transition to death state after surgery. In addition, tumor size had significant effect on transition of the first recurrence of tumor state to death state ^28^. Tumor size, human epidermal growth hormone receptor status and proportion of lymph nodes were sectoring prognostic risk factors for free BC survival using recurrent events and Anderson-Gill multiple models ^29^. Age, lymph nodes, tumor grade and ER status to be significantly associated with hazard of death of breast cancer patients ^15^. Putter et al predicted trend of BC using multi-state model. They defined disease topical relapse status, distant metastasis, topical relapse status, simultaneous distant metastasis and death as different states of the disease. According to the influence of prediction factors, they used estimated transition probabilities between two states of disease for predicting the disease process. Patients with tumor size greater than 5 cm had 1.2 times more risk of recurrence of BC compared to patients with tumor size less than 2 cm ^14^. The age at diagnostic had a significant effect on the risk of death in patients in patients without recurrence of BC using multi-state model, but tumor size had no significant effect on the occurrence of the first recurrence ^30^.

In this study, age, tumor size, the fraction of the number of involved lymph nodes to the number of removed lymph nodes, HER2, P53 were affected on transitions of states. In addition, recovery for a patient who was in the initial state was more than other states.

The time that disease remains in the preclinical detectable phase, the sojourn time, is important, especially in a screening program ^31^. In this sense, our results showed that the maximum mean sojourn time, considering the influence of independent variables was related to recovery state.

## Conclusion

The multi-state model with independent variables was better fitted than model without independent variable. In this model, the hazard ratios at different times can be estimated using transition intensity and probability matrix. Although, interpretation of some of the estimated hazard ratio for different transitions may not be clinically valuable; but the process of disease for patients with these characteristics and other entered variables can be predicted and then therapeutic actions can be suggested and performed. On the other hand, with estimated transition probabilities and also survival mean in each state, the physicians will be able to suggest appropriate care and/or treatment for patients.

## Acknowledgements

Thanks to Seyed-o-Shohada Hospital staff and Isfahan Cancer Prevention Research Center to helped us on data gathering. This study was accepted, IR.USWR.REC.1396.97, and supported by the Deputy of Research and Technology of University of Social Welfare and Rehabilitation Sciences in Tehran, Iran

## Conflict of interest

The authors declare that they have no conflicting interests.

## Funding

This study was supported by the Deputy of Research and Technology of University of Social Welfare and Rehabilitation Sciences in Tehran, Iran.

## Highlights

- A patient who was in the initial state of treatment had more transition probability to recover state than other states, in a 5-year period of time.
- The maximum mean sojourn time was related to recovery state, equal to 6.93 years.
- The hazard ratios for each risk factor and in each transition path were calculated. As an example, with increasing tumor size, the risk of death for those in initial state treatment was more than other states.
